# Correction: Li et al. Gastrodin Ameliorates Cognitive Dysfunction in Vascular Dementia Rats by Suppressing Ferroptosis via the Regulation of the Nrf2/Keap1-GPx4 Signaling Pathway. *Molecules* 2022, *27*, 6311

**DOI:** 10.3390/molecules31122099

**Published:** 2026-06-15

**Authors:** Yue Li, Erdong Zhang, Hong Yang, Yongxin Chen, Ling Tao, Yini Xu, Tingting Chen, Xiangchun Shen

**Affiliations:** 1 Guiyang Maternal and Child Health-Care Hospital, Guiyang 550002, China; 2 The State Key Laboratory of Functions and Applications of Medicinal Plants, Guizhou Medical University, University Town, Guiyang 550025, China; 3 The High Efficacy Application of Natural Medicinal Resources Engineering Center of Guizhou Province, The High Educational Key Laboratory of Guizhou Province for Natural Medicinal Pharmacology and Druggability, Guizhou Medical University, University Town, Guiyang 550025, China

**Error in Figure 2:**

In the original publication [1], there was a mistake in Figure 2F as published. The protein bands of Figure 2F were misplaced during the preparation of Figure 2. The corrected Figure 2F shows the protein band of COX2, not the protein band of GPx4, as displayed below. Also, References 10, 19 and 37 have been replaced. We apologize for any inconvenience caused and state that the scientific conclusions are unaffected. This correction was approved by the Academic Editor. The original publication has also been updated.

**Figure 2:**
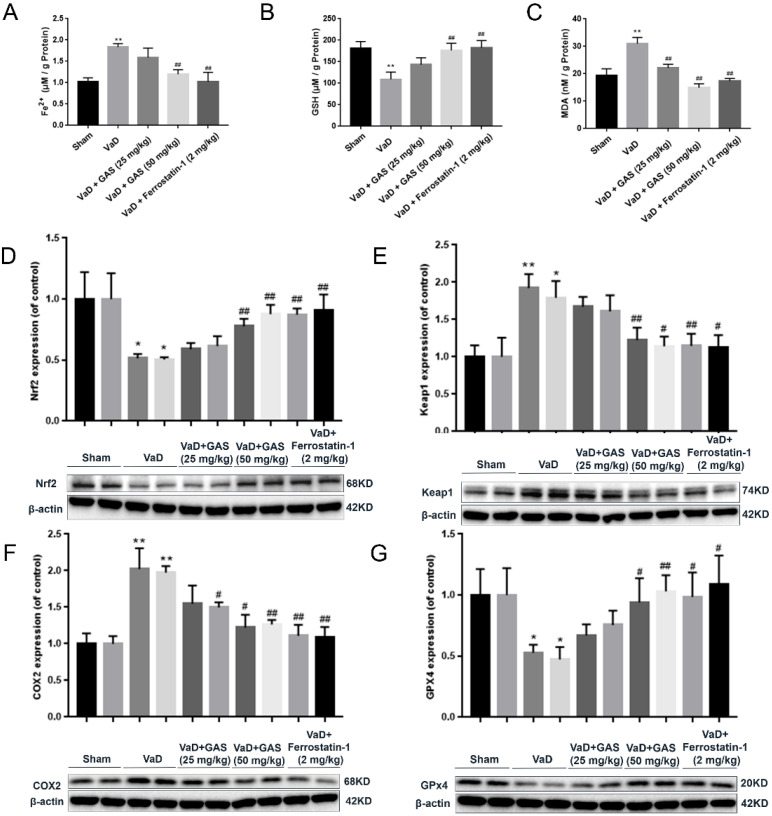
Effects of GAS on ferroptosis indexes in the hippocampus of VD rats. (**A**–**C**), detection of biochemical indicators of ferroptosis, (**D**–**G**), Western blot analyses of ferroptosis-related protein expression. Results are shown as mean ± SD (*n* = 6). * *p* < 0.05, ** *p* < 0.01 specifies the differences between sham rat and VaD rat. ^#^ *p* < 0.05, ^##^ *p* < 0.01 compares between VaD rat and GAS-treated rat or ferrostatin1-treated rat.

## Revised References

Reference 19 has been retracted, and references 10 and 37 have been replaced:

10. Tuo, Q.Z.; Zhang, S.T.; Lei, P. Mechanisms of neuronal cell death in ischemic stroke and their therapeutic implications. *Med. Res. Rev.* **2022**, *42*, 259–305.
19. Tang, J.J.; Huang, L.F.; Deng, J.L.; Wang, Y.M.; Guo, C.; Peng, X.N.; Liu, Z.; Gao, J.M. Cognitive enhancement and neuroprotective effects of OABL, a sesquiterpene lactone in 5xFAD Alzheimer’s disease mice model. *Redox. Biol.* **2022**, *50*, 102229.
37. Sohn, E.; Kim, Y.J.; Lim, H.S.; Kim, B.Y.; Jeong, S.J. Hwangryunhaedok-Tang exerts neuropreventive effect on memory impairment by reducing cholinergic system dysfunction and inflammatory response in a Vascular Dementia rat model. *Molecules* **2019**, *24*, 343.
