# Supplementary material for: Gastrodin Ameliorates Cognitive Dysfunction in Vascular Dementia Rats by Suppressing Ferroptosis via the Regulation of the Nrf2/Keap1-GPx4 Signaling Pathway
Source: Molecules. 2022 Sep 24;27(19):6311. doi: 10.3390/molecules27196311 (PMC9571513; doi:10.3390/molecules27196311)
Supplement: Supplementary file 1 [file molecules-27-06311-s001.zip › molecules-1912157-supplementary.pdf]

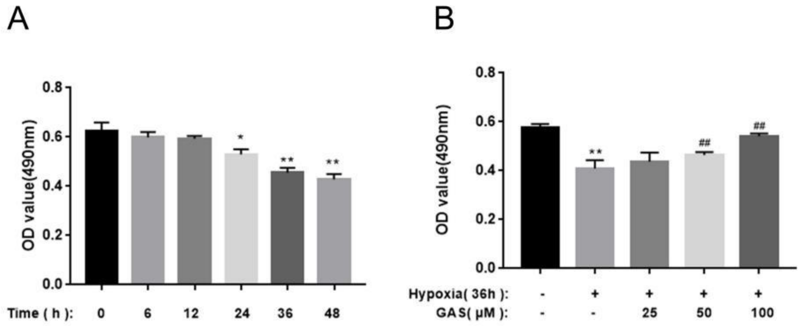

Figure S1. The protective effect of gastrodin on hypoxia-induced injury of HT22 cells. (A) MTT was used to detect the survival rate of HT22 cells after hypoxia for 0h, 6h, 12h, 24h, 36h and 48h; (B) MTT assay was used to detect the protective effect of gastrodin on hypoxia-induced injury of HT22 cells. Results are shown as mean ± SD. $^{*}p < 0.05$, $^{**}p < 0.01$ versus control, $^{\#}p < 0.05$, $^{\#\#}p < 0.01$ versus hypoxia.
